# Correction: Maskato et al. Prospective Feasibility and Revalidation of the Equine Acute Abdominal Pain Scale (EAAPS) in Clinical Cases of Colic in Horses. *Animals* 2020, *10*, 2242

**DOI:** 10.3390/ani12081001

**Published:** 2022-04-13

**Authors:** Yamit Maskato, Alexandra H. A. Dugdale, Ellen R. Singer, Gal Kelmer, Gila A. Sutton

**Affiliations:** 1 Large Animal Department, Robert H Smith, Faculty of Agriculture, Food and Environmental Sciences, Koret School of Veterinary Medicine, Veterinary Teaching Hospital, The Hebrew University of Jerusalem, P.O. Box 12, Rehovot 7610001, Israel; yamit.maskato@mail.huji.ac.il (Y.M.); gal.kelmer@mail.huji.ac.il (G.K.); 2 Leahurst Campus, School of Veterinary Science, University of Liverpool, Chester High Road, Neston CH64 7TE, UK; alexdugdale@btinternet.com (A.H.A.D.); singer844@btinternet.com (E.R.S.)

The authors wish to make the following corrections to this paper [1]:

In the original published article, the Institutional Review Board Statement was not included. The Institutional Review Board Statement is added below.

**Institutional Review Board Statement:** Ethics approval was obtained from each of the two institutions: The Internal Ethics Review Committee of the Koret School of Veterinary Medicine Veterinary Teaching Hospital, Israel, certificate number KSVM-VTH/07_2015 and Veterinary Research Ethics Committee, University of Liverpool, UK, certificate number VREC237.

The authors apologize for any inconvenience caused and state that the scientific conclusions are unaffected. The original publication has also been updated.
