# Supplementary material for: Prospective Feasibility and Revalidation of the Equine Acute Abdominal Pain Scale (EAAPS) in Clinical Cases of Colic in Horses
Source: Animals (Basel). 2020 Nov 29;10(12):2242. doi: 10.3390/ani10122242 (PMC7760242; doi:10.3390/ani10122242)
Supplement: Supplementary file 1 [file animals-10-02242-s001.pdf]

animals

MDPI

Questionnaire for Users of

The Equine Acute Abdominal Pain Scale (EAAPS)

Case no.: ________________ **Date:** ___________________ **Time:** ___________________

Assessor's D.O.B.: ______________________

Clinician to complete on admission of the horse / pony

(Final pain score is the highest scoring-behaviour)

	O	A	B	C	D	E
**Equine Acute Abdominal Pain Score (EAAPS)**	No overt signs of pain	Flank watching<br>Lip curling	Sternal recumbency<br>Stretching<br>Restlessness	Kicking belly<br>Pawing ground	Attempting to lie down<br>Lateral recumbency	Rolling

Please fill in the blanks by choosing the most appropriate word;

TIME:

1. I was able to score this horse ____________;

Very quickly   Quickly   Within a reasonable time   Slowly   Very slowly

EFFORT:

2. It was _______________ to achieve an EAAPS score for this horse;

Very Easy   Easy   Not Easy/Not Difficult   Difficult   Very Difficult

FAMILIARITY WITH SCORING SYSTEM:

3. This is the _______________ time that I have used the EAAPS score;

First   Second   Third   Greater than third   Lost count/can't remember

SCORE:

4. (Optional) If I could, I would change the EAAPS by:

____

____

____

Figure S1. The Equine Acute Abdominal Pain Scale form used in the UK for data collection. This form was attached to the standard admission forms for cases of colic. The visual analogue scale was embedded in the form in its location prior to this study.

1

Questionnaire for Users of The Equine Acute Abdominal Pain Scale (EAAPS)

Please complete this entire form either when the horse demonstrates **pain for the first time** or **before administrating analgesia** if the horse does not show any pain.

Case no.: ________________**Date**: ____________________**Time**: ___________________

***Assessor's name or date of birth**: _______________________

*Please be consistent with your choice.

Mark one: DVM _____ Student _____ Technician _____ Owner _____

Location of horse: stocks ______ stall ________ paddock or roundpen ________

Prior analgesia: analgesic ____________ dose_________ administered when?_________

**Please mark a cross on the line below so that the distance from the "pain free" side to the mark represents the pain score.**
Pain free = 0———————————————————— 10 = Severe pain

Pain score = number of mm along the line from the left hand end to the mark

IMPORTANT: Please TURN OVER THE PAGE

Figure S2. The Equine Acute Abdominal Pain Scale form used in Israel for data collection. a) page 1; b) page 2; S2a).

2

PLEASE BEGIN ON OPPOSITE SIDE OF THE PAGE

Please circle any of the behaviours below that the horse is demonstrating. The ***final Pain Score*** *is the letter that applies to the behaviour associated with the cell that is* ***farthest to the right***.

	O	A	B	C	D	E
**Equine Acute Abdominal Pain Score (EAAPS)**	No overt signs of pain	Flank watching<br>Lip curling	Sternal recumbency<br>Stretching<br>Restlessness	Kicking belly<br>Pawing ground	Attempting to lie down<br>Lateral recumbency	Rolling

<u>Please fill in the blanks by choosing the most appropriate word</u>;

TIME:

1. I was able to score this horse _____________;

   Very quickly Quickly Within a reasonable time Slowly Very slowly

EFFORT:

2. It was ________________ to achieve an EAAPS score for this horse;

   Very Easy Easy Not Easy/Not Difficult Difficult Very Difficult

FAMILIARITY WITH SCORING SYSTEM:

3. This is the ________________ time that I have used the EAAPS score;

   First Second Third Greater than third Lost count/can't remember

SCORE:

4. (Optional) If I could, I would change the EAAPS by:

   ____

   ____

Figure S2. The Equine Acute Abdominal Pain Scale form used in Israel for data collection. a) page 1; b) page 2; S2b.

Publisher's Note: MDPI stays neutral with regard to jurisdictional claims in published maps and institutional affiliations.

© 2020 by the authors. Licensee MDPI, Basel, Switzerland. This article is an open access article distributed under the terms and conditions of the Creative Commons Attribution (CC BY) license (http://creativecommons.org/licenses/by/4.0/).
